# Comparative study on the effects of glutamic acid and glutamine in promoting intestinal development in chicks through energy metabolism

**DOI:** 10.5713/ab.25.0445

**Published:** 2025-09-30

**Authors:** Peiyu Huang, Yaoming Cui, Wenjing Liang, Li Zhang, Junquan Tian, Liping Gan, Linna Guo, Weiyu Chen, Guohao Yang, Junjun Guan

**Affiliations:** 1 School of Biological Engineering, Henan University of Technology, Zhengzhou, China

**Keywords:** Energy Metabolism, Glutamic Acid, Glutamine, Intestinal Development, Intestinal Organoid, Layer Chick

## Abstract

**Objective:**

This study evaluated the effects of glutamic acid (Glu) and glutamine (Gln) on the intestinal development of layer chicks with lipopolysaccharide (LPS)-induced damage.

**Methods:**

A total of 240 healthy 0-d-old Hy-Line Brown chicks were randomly assigned to 4 treatments, each with 6 replicates. At 8 and 11 d of age, all birds (except for the control group) received two administrations of LPS. The LPS-challenged birds were divided into three dietary treatment groups: a basal diet (without additives), a 0.05% Glu-supplemented diet, and a 0.20% Gln-supplemented diet.

**Results:**

The LPS challenge induced intestinal injury and suppressed intestinal development in layer chicks, as evidenced by reduced growth performance, poor intestinal parameters, and morphology (p<0.05). Compared to the LPS group, dietary supplementation with 0.05% Glu and 0.20% Gln enhanced average daily gain (ADG), average daily feed intake, body weight (BW), and intestinal development parameters (including length, weight, villus height, and villus height/crypt depth) of duodenum, jejunum and ileum (p<0.05). These results could be attributed to upregulated mRNA expression levels of *Mucin-2*, *E-cadherin*, *Dclk-1*, *Vil-1*, *Lysozyme*, *ChgA*, *Lgr-5*, *Bmi-1*, *ATP5F1AZ*, and *β-catenin* (p<0.05). Furthermore, dietary supplementation with 0.20% Gln outperformed 0.05% Glu in enhancing BW, ADG, and ileum parameters (weight, length, epithelial cell count, and energy metabolism) (p<0.05). Additionally, intestinal organoids supplemented with 10 μM Gln had higher mean area, *E-cadherin* gene expression, and ATP content compared with those treated with 5 μM Glu *in vitro* (p<0.05).

**Conclusion:**

Dietary supplementation with 0.05% Glu and 0.20% Gln could improve growth performance, intestinal development, and repair intestinal damage in layer chicks through enhanced epithelial proliferation and differentiation. Moreover, 0.20% Gln performed better than 0.05% Glu, which may be attributed to superior energy metabolism.

## INTRODUCTION

The laying chicken industry plays a crucial role in agricultural production, and China’s egg production reached 35.88 million tons in 2024. Impaired early intestinal development in laying chickens can cause irreversible damage to their subsequent growth and production. An insufficient energy supply is considered one of the most important reasons [1]. Upon hatching, the chick’s intestinal development is still immature, which limits its ability to digest feed and utilize nutrients [2], which are derived from the residual yolk and absorbed via the intestines [3]. However, this absorption process is too slow to meet the high energy demands of rapid intestinal development. Furthermore, after hatching, the chick’s small intestine undergoes morphological, cellular, and molecular modifications to prepare for the switch to an external food source [4], a process that is highly stressful and energy-consuming. In conclusion, promoting intestinal development and maintaining intestinal barrier integrity require a substantial energy supply. Thus, identifying appropriate exogenously added energy substrates is crucial for the early intestinal development of layer chicks.

Glutamine (Gln) and glutamic acid (Glu), which are supposed to be primary energy substrates for the rapidly renewing intestinal epithelium [5,6], have been demonstrated to aid in repairing intestinal damage [7,8]. However, it has also been observed that higher-than-optimal dosages of these nutrients do not provide additional benefits to intestinal health and may even suppress growth performance and intestinal development [9,10]. Thus, further research is required to investigate the appropriate dosages of Gln and Glu for energy supply in layer chicks.

In fact, Gln and Glu, as interconvertible amino acids, are involved in ATP synthesis through the tricarboxylic acid cycle, but their energy-supplying efficacy may vary depending on physiological factors and developmental stages [11,12]. Gln is recognized as a conditionally essential amino acid and can be utilized to meet increased energy demands under stress conditions [13]. In contrast, no reports suggest that Glu possesses similar properties under stress conditions. Moreover, the differences between these 2 amino acids in the intestinal development of chicks remain insufficiently clarified [14], particularly due to the severe lack of systematic comparative research in layer chicks.

Therefore, the present study aimed to compare the effects of Glu and Gln on promoting intestinal development and alleviating injury in layer chicks using both *in vivo* and *in vitro* models. The impacts of dietary supplementation with Glu and Gln on growth performance, intestinal development, intestinal morphology, and energy metabolism were investigated *in vivo*. Additionally, the impacts of supplemented Glu and Gln on the growth-related gene abundances, development, and energy metabolism were evaluated in intestinal organoids.

## MATERIALS AND METHODS

### Birds and experimental design

A total of 240 healthy 0-d-old Hy-Line Brown chicks were randomly assigned to 4 treatments, each with 6 replicates (10 birds per replicate). Except for the control group, all birds were injected with LPS (1 mg/kg body weight [BW], Solarbio) twice in the abdomen at 8 and 11 d of age. The LPS-challenged birds were divided into three dietary treatment groups: basal diet (without additives), 0.05% Glu-supplemented diet, and 0.20% Gln-supplemented diet, with Glu and Gln sourced from Macklin Biochemical Technology. Our unpublished data (Supplements 1–4) indicated that optimal growth performance and intestinal development occurred with 0.05% Glu and 0.20% Gln supplementation, respectively. Therefore, these dosages were selected for this investigation. The housing area and feeding apparatus were meticulously cleaned and sanitized in strict compliance with the recommended methodology before the experiment’s commencement. Regular control of the ventilation system and prompt rubbish collection ensured the air purity of the housing area. The housing area was cleaned regularly. Before introducing the layer chicks, the housing area temperature was increased to 33°C and decreased by 3°C each week until it reached 27°C. Water and feed were readily available throughout the trial. Formulated according to the NRC [15] and the Chinese Feeding Standard of Chicken (NY/T 33-2004) [16], the experimental diets were prepared. The basic diet composition and nutrient level were displayed in Table 1 during the 3-week experiment.

### Growth performance and sample collection

The BW of layer chicks was measured at 0, 7, 14, and 21 d in this study, and the amount of feed consumed each week was recorded. The average daily gain (ADG), average daily feed intake (ADFI), and feed conversion ratio (FCR, feed intake/BW gain, g:g) were calculated. The time of death, feed allowance, and weight of birds were noted once the death of a chick was observed. At 7, 14, and 21 d of age, 2 chicks (at average BW) were chosen randomly from 6 replicates in each group and weighed before slaughter. To investigate the effects of dietary supplementation with appropriate levels of Glu and Gln on intestinal development, the weight and length of the duodenum, jejunum, and ileum were measured.

Approximately 1.5 cm sections of the duodenum, jejunum, and ileum were obtained after slaughter and washed with phosphate-buffered saline (PBS, Solarbio), preserved in 4% neutral buffered paraformaldehyde for intestinal morphology sectioning and microscopic analysis using hematoxylin and eosin (H&E) staining. To evaluate the mRNA expression of target genes (Supplement 5), mucosal samples were obtained by opening the remaining ileum sections along their length. These samples were then rapidly submerged and stored in liquid nitrogen.

### Intestinal morphology

The samples from 3 intestinal segments were embedded, stained, and viewed under a biological microscope (Ningbo Sunny Instrument) according to the published method [17]. The corresponding average of each chick was recorded as the mean of all measurements per sample. The intestinal sections were evaluated for morphology utilizing villus height (VH), crypt depth (CD), and the villus height to crypt depth ratio (VCR) [18].

### Quantification of mRNA with real-time polymerase chain reaction

The FreeZol Reagent (Vazyme) was utilized to extract total RNA from organoids and mucosa samples following the manufacturer’s instructions. A microvolume spectrophotometer (Keen Innovative Solutions) was used to measure the yield and purity of RNA, and the A260/A280 ratio exceeded 1.8. The RNA was stored at −80°C for subsequent cDNA synthesis. Following the manufacturer’s instructions, a reverse transcription kit (Vazyme) was used to convert the extracted total RNA into cDNA. The qTOWER 3G real-time fluorescence quantitative polymerase chain reaction (qPCR) equipment (Analytik Jena) was used to perform real-time fluorescence quantitative PCR using a Taq SYBR Green qPCR Premix kit. The procedure included an initial denaturation step at 95°C for 30 s. Following this step were 40 cycles of denaturation (at 95°C for 15 s each) and annealing and extension (each lasting 30 s at 60°C). The primer sequences of the investigated genes are listed in Supplement 6 and were produced by Shanghai Shenggong Bioengineering, some of which are referenced from earlier reports [19,20]. *β-actin* was used as the internal reference gene, and the 2^−ΔΔCT^ method was utilized for the analysis and comparison of the mRNA relative expression levels.

### Energy metabolism

The ATP content, Na^+^-K^+^-ATPase activity, and Ca^2+^-Mg^2+^-ATPase activity in the intestinal mucosa and organoids were determined using a colorimetric method according to the published study [21]. These assays were performed using kits (A095-1-1 and A016-2) from Nanjing Jiancheng Bioengineering Institute.

### Isolation and culture of intestinal crypts

**Figure 1:**
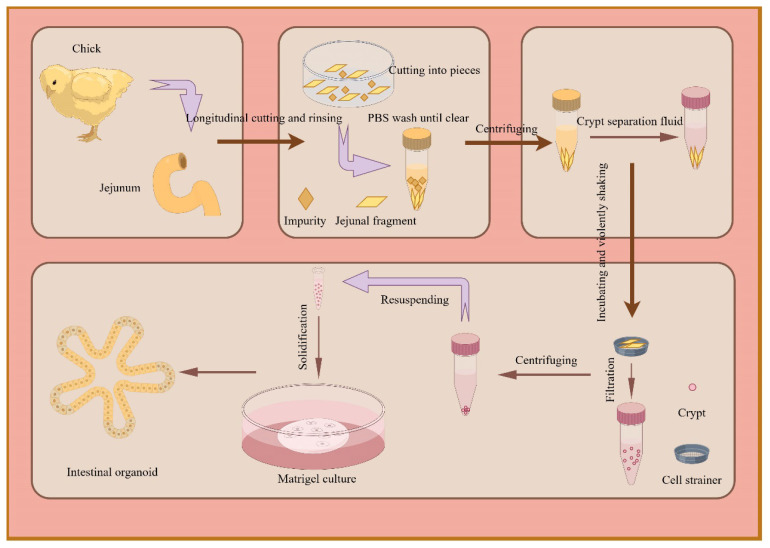
The general procedure for intestinal crypt isolation and culture of layer chicks.

The crypt separation and culture method was established after minor modifications based on previous reports [19,22], and the general process is shown in Figure 1. The jejunum segment from 7-d-old layer chicks is placed in a cell culture dish and set on ice. After the intestinal segment was cut longitudinally and washed with PBS, it was divided into pieces (0.3–0.5 cm) and gently agitated. The supernatant was then removed and replaced with a cold solution of Dulbecco’s PBS (DPBS; Solarbio), which included 5 mM ethylenediaminetetraacetic acid (EDTA; Solarbio). Following incubation, the mixture was shaken vigorously and filtered through 100 and 75 μm cell strainers, and purified crypts were obtained. Afterward, the organoid growth medium (OGM; the manufacturing method is detailed in Supplement 6) and Matrigel were added and mixed thoroughly to resuspend the purified crypts, which were then cultured in an incubator at 37°C with 5% CO_2_. Inverted microscopy was used to obtain live images of intestinal organoids (Cossim).

To investigate the effects of energy substrates on organoid growth, the established intestinal organoids were cultured in OGM supplemented with a range of concentrations (5, 10, 100, and 1,000 μM) of both Glu and Gln to identify the optimal concentrations of these 2 energy substrates, respectively. In addition, detailed methods for 5-ethynyl-2′-deoxyuridine (EdU) labeling and immunofluorescence, designed to elucidate the morphological structure of the established organoid model, are provided in Supplement 7.

### Statistical analysis

The replicate, each in 1 cage, serves as the growth performance analysis experimental unit. The experimental unit used to measure and analyze other parameters was two chicks per replication. For data analysis, ver. 9.2 of the software from SAS Institute, was utilized. First, the homogeneity of variances and data normality were examined, and the Shapiro-Wilk test was used to assess normality. After that, a one-way ANOVA was performed, and Tukey’s Multiple Comparison Test was applied to compare the means. p*<*0.05 was used to determine statistical significance for differences, and the results were presented as mean and pooled standard error of the mean (SEM).

## RESULTS

### Impact of glutamic acid and glutamine on the growth performance of layer chicks

The impact of dietary supplementation with 0.05% Glu and 0.20% Gln on the growth performance of layer chicks is presented in Table 2. No differences in BW were noted at the beginning of the trial among all treatment groups of layer chicks. Between the control and LPS groups, no differences were observed before LPS injection (0 to 7 d). The BW values in the Glu and Gln groups were greater than those in the control and LPS groups at 7 d, and a considerably higher value of ADG (0 to 7 d) was noted in the Gln group (p*<*0.05). During the LPS challenge (7 to 21 d), ADG (7 to 14 d, 14 to 21 d, 0 to 21 d), ADFI (7 to 14 d, 0 to 21 d), BW (14 and 21 d), and FCR (7 to 14 d, 0 to 21 d) were all lower in the LPS group than in the control group (p*<*0.05). Following LPS injection, higher values of the BW (14 and 21 d), ADG (7 to 14 d, 0 to 21 d), ADFI (7 to 14 d, 0 to 21 d), and FCR (7 to 14 d, 14 to 21 d, 0 to 21 d) were observed in the Glu and Gln groups compared with the LPS group. Furthermore, ADG (14 to 21 d) was higher in the Gln group than in the control and LPS groups (p*<*0.05), whereas it was only higher in the Glu group than in the LPS group. Meanwhile, a higher value of ADG (0 to 21 d) was observed in the Gln treatment compared with the Glu treatment (p*<*0.05).

### Impact of glutamic acid and glutamine on the small intestine injury repair of layer chicks

The effects of dietary supplementation with 0.05% Glu and 0.20% Gln on the development of the duodenum, jejunum, and ileum in layer chicks are depicted in Table 3. The parameters in the LPS and control groups were not different before LPS administration. In terms of jejunum (weight and index), ileum (weight, index, and length), and total values (weight and index), the Glu and Gln groups performed better at 7 d than the control and LPS groups (p<0.05). Furthermore, the length of the duodenum and jejunum was longer in the Gln group than in the control and LPS groups (p*<*0.05). After the LPS challenge, lower values of the duodenum (weight, 14 and 21 d), jejunum (weight, index, and length, 14 and 21 d), ileum (weight and index, 14 d; weight and length, 21 d) and total values (weight and index, 14 and 21 d) were observed in the LPS group compared with the control group (p*<*0.05). Except for the duodenum (index, 14 and 21 d), ileum (index, 21 d), and total value (index, 21 d), all parameters enhanced in the Glu and Gln treatments in comparison to the LPS group after receiving LPS administration (p*<*0.05). The duodenum (weight, 21 d), jejunum (weight, 21 d), ileum (weight and index, 14 and 21 d; length, 14 d), and total values (weight and index, 21 d) were substantially greater in the Gln group than in the control group. However, compared to the control group, only the jejunum (weight, 14 and 21 d; length, 21 d) showed a increase in the Glu group (p*<*0.05). Furthermore, the duodenum (weight and index, 21 d), jejunum (weight and index, 21 d), ileum (weight and index, 14 and 21 d; length, 21 d), and total values (weight and index, 21 d) increased in the Gln group compared with the Glu group (p*<*0.05).

### Impact of glutamic acid and glutamine on the intestinal morphology of layer chicks

**Figure 2:**
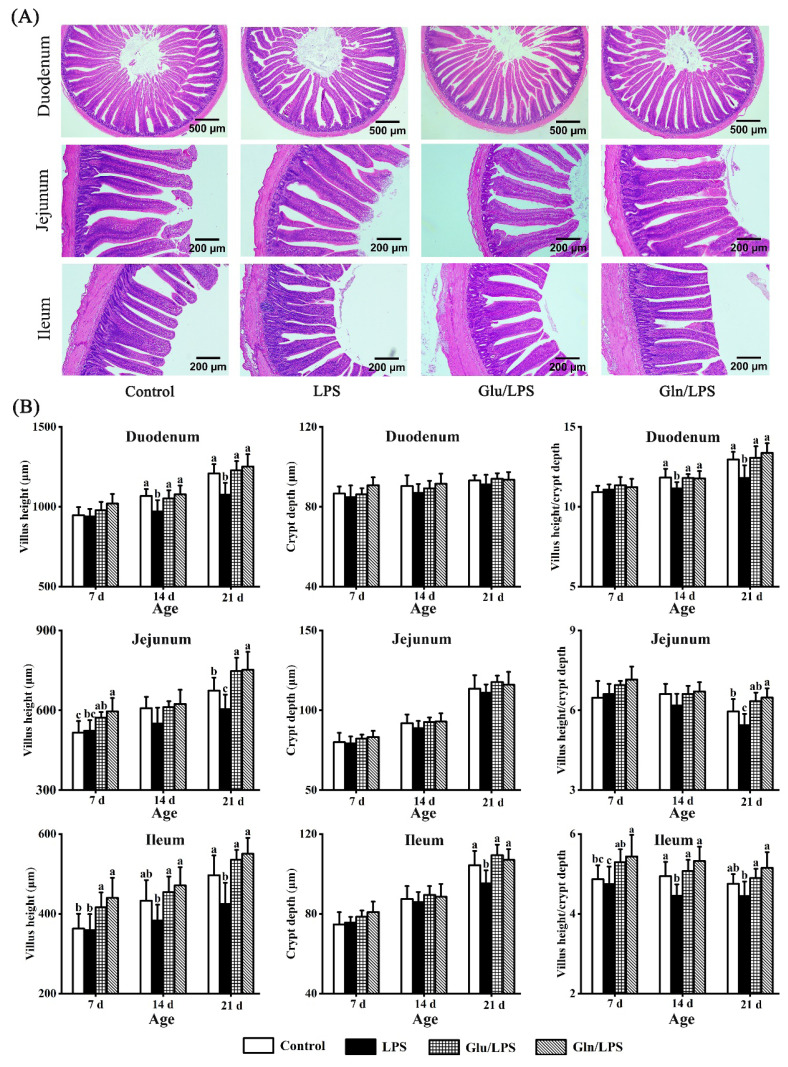
Impact of dietary supplementation with Glu and Gln on the intestinal morphology of layer chicks (Hematoxylin and eosin staining). Control = fed the basal diet; LPS = fed the basal diet and received lipopolysaccharide (LPS) administration; Glu/LPS = fed the basal diet supplemented with 0.05% glutamic acid and received LPS administration; Gln/LPS = fed the basal diet supplemented with 0.20% glutamine and received LPS administration. The mean of 6 replicates is used as the data. ^a–c^ Without any common superscripts, values amongst groups on the same day of age differ significantly (p<0.05), and the standard deviation (SD) is shown by the error bars. Glu, glutamic acid; Gln, glutamine.

As shown in typical intestinal longitudinal sections, the intestinal morphology in Glu/LPS and Gln/LPS treatments was superior to that observed in the LPS treatment (Figure 2A). Figure 2B displays the modifications in intestinal morphology resulting from 0.05% Glu and 0.20% Gln supplementation in the diet. Prior to the LPS injection (7 d), there was no discernible difference in the parameters between the control and LPS groups. Meanwhile, the greater values of the VH (ileum and jejunum) were noted in the Glu and Gln groups in comparison to the control group (p*<*0.05). Furthermore, the Gln group had a higher VCR of the ileum compared with the control and LPS groups (p*<*0.05). Except for the CD of the ileum at 21 d, there were no discernible variations in the CD of the intestine among the groups. After LPS injection (14 and 21 d), the lower value of all parameters except for CD (duodenum and jejunum, 14 and 21 d; ileum, 14 d), VH (ileum and jejunum, 14 d), and VCR (jejunum, 14 d; ileum, 21 d) were observed in the LPS group in comparison to the control group (p*<*0.05). Otherwise, the VH (ileum, 14 and 21 d; jejunum, 21 d) increased in the Glu and Gln groups compared with the control group (p*<*0.05). Concurrently, compared to the LPS group, larger values of all parameters except for intestinal CD (14 and 21 d) and jejunum (VH and VCR, 14 d) were observed in the Glu and Gln groups compared with the control group (p*<*0.05). However, no discernible difference was observed in parameters between the Glu and Gln groups.

### Impact of glutamic acid and glutamine on the gene abundances in the ileum of layer chicks

**Figure 3:**
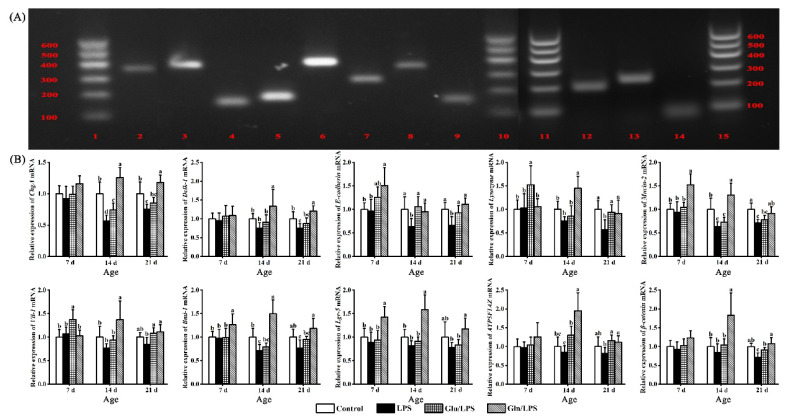
The effects of dietary supplementation with 0.05% Glu and 0.20% Gln on the gene abundances in the ileum of layer chicks. (A) A representative image of RT-PCR results from lane 1 to lane 15 are, DNA ladder (100, 200, 300, 400, 500 and 600 bp), *ChgA* (337 bp), *Mucin-2* (357 bp), *Vil-1* (141 bp), *ATP5F1AZ* (180 bp), *β-catenin* (374 bp), *Bmi-1* (255 bp), *Lgr-5* (338 bp), *β-actin* (150 bp), DNA ladder, DNA ladder, *Dclk-1* (196 bp), *E-cadherin* (226 bp), *Lysozyme* (71 bp), DNA ladder, respectively. (B) The mRNA relative expression of intestinal functional epithelial cells, stem cells marker genes, and energy metabolism-related genes in layer chicks. The mean of 6 replicates is used as the data. ^a–c^ Without any common superscripts, values amongst groups on the same day of age differ significantly (p<0.05), and the standard deviation (SD) is shown by the error bars. Glu, glutamic acid; Gln, glutamine; PCR, polymerase chain reaction.

To confirm the specificity of the RT-PCR reaction, a representative image of RT-PCR results is provided (Figure 3A). The gene abundances of the selected genes in the ileum are depicted in Figure 3B. Compared to the control and LPS treatments, the Gln treatment had increased gene abundances of *E-cadherin*, *Mucin-2*, *Bmi-1*, and *Lgr-5* before the LPS injection (7 d), and enhanced gene abundances of *Lysozyme*, *Vil-1*, and *Bmi-1* were noted at 7 d in the Glu treatment (p*<*0.05). Following LPS injection (14 and 21 d), the mRNA relative expressions of *ChgA*, *E-cadherin*, *Mucin-2*, and *Bmi-1* at 14 d, and those of *Dclk-1*, *Lysozyme*, and *β-catenin* at 21 d dropped in the LPS treatment compared with the control treatment (p*<*0.05). Meanwhile, the Glu treatment showed substantially enhanced gene abundances of *ChgA*, *E-cadherin*, and *ATP5F1AZ* at 14 d, as well as *E-cadherin*, *Lysozyme*, *Vil-1*, and *β-catenin* at 21 d compared with the LPS treatment (p*<*0.05). However, compared to the LPS treatment, substantially higher values of target gene abundances were observed in the Gln treatment at 14 and 21 d (p*<*0.05). Moreover, higher values of target gene abundances at 14 d, except for *E-cadherin*, and those of *ChgA*, *Dclk-1*, *β-catenin*, *Bmi-1*, and *Lgr-5* at 21 d were observed in the Gln treatment compared with the Glu treatment (p*<*0.05).

### Effects of glutamic acid and glutamine supplementation on the energy metabolism in the small intestinal mucosa of layer chicks

The impact of 0.05% Glu and 0.20% Gln supplementation in the diet on energy metabolism in the small intestinal mucosa of layer chicks is presented in Table 4. No differences were observed among the treatment groups in the parameters of the duodenum. Compared to the control group, lower values of ATP content (ileum), Na^+^-K^+^-ATPase activity (jejunum and ileum), and Ca^2+^-Mg^2+^-ATPase activity (jejunum) were observed in the LPS group (p**<**0.05). Furthermore, higher values of ATP content, Na^+^-K^+^-ATPase activity, and Ca^2+^-Mg^2+^-ATPase activity were observed in the jejunum and ileum of layer chicks in the Glu and Gln groups compared with the LPS group (p**<**0.05). Additionally, the ATP content, Na^+^-K^+^-ATPase activity, and Ca^2+^-Mg^2+^-ATPase activity of the ileum increased in the Gln group compared with the Glu group (p**<**0.05), and no differences were observed in the remaining parameters.

### Impact of different glutamic acid and glutamine concentrations on the development of intestinal organoids

**Figure 4:**
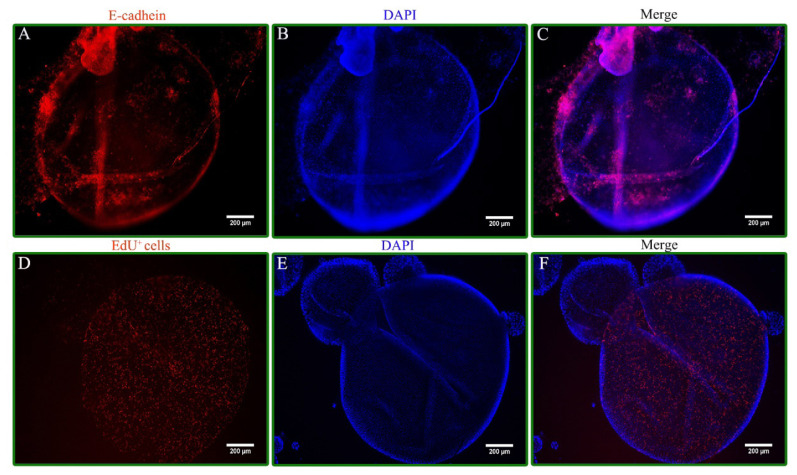
The representative images of immunofluorescence and EdU staining at 5 d of organoid culture. Scale bar, 200 μm. DAPI, 4′,6-diamidino-2-phenylindole; EdU, 5-ethynyl-2′-deoxyuridine.

**Figure 5:**
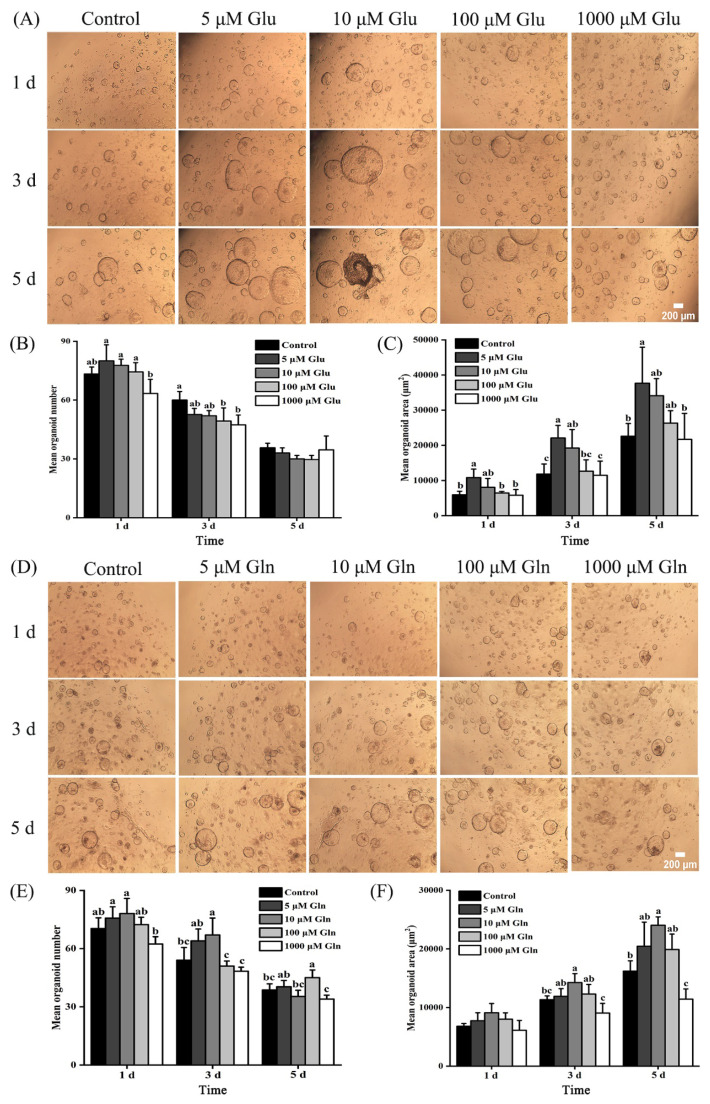
Effects of Glu and Gln supplementation in the OGM on the development of intestinal organoids. The mean of 3 replicates was used as the data. ^a–c^ Without any common superscripts, values amongst groups on the same day of age differ significantly (p<0.05), and the standard deviation (SD) is shown by the error bars. Glu, glutamic acid; Gln, glutamine; OGM, organoid growth medium.

Representative images of immunofluorescence and EdU staining from the intestinal organoid model implied that the organoids had a lumen structure and intestinal stem cells located within the lumen proliferated and differentiated into epithelium (Figure 4). As shown in Figures 5A–5C, the mean organoid area enhanced in the 5 μM Glu treatment compared with the control group at 1, 3, and 5 d (p<0.05), however, no discernible changes were noted in the mean organoid number at 1, 3, and 5 d. Furthermore, lower values of the mean organoid number (1 d) and the mean organoid area (1, 3, and 5 d) were noted in the 1,000 μM Glu treatment compared with the 5 μM Glu treatment (p<0.05). However, no substantial changes were noted in the mean organoid number at 3 and 5 d (Figures 5A–5C). As shown in Figures 5D–5F, compared to the control group, there were no changes in the mean organoid number at 1 and 5 d in the 10 μM Gln treatment. However, the mean organoid number (3 d) and the mean organoid area (3 and 5 d) enhanced in the 10 μM Gln treatment (p<0.05). Additionally, compared to the 10 μM Gln treatment, the mean organoid number (1 and 3 d) and the mean organoid area (3 and 5 d) decreased in the 1,000 μM Gln treatment (Figures 5D–5F; p<0.05).

### Impact of appropriate glutamic acid and glutamine dosages on the development, gene expression and energy metabolism of intestinal organoids

**Figure 6:**
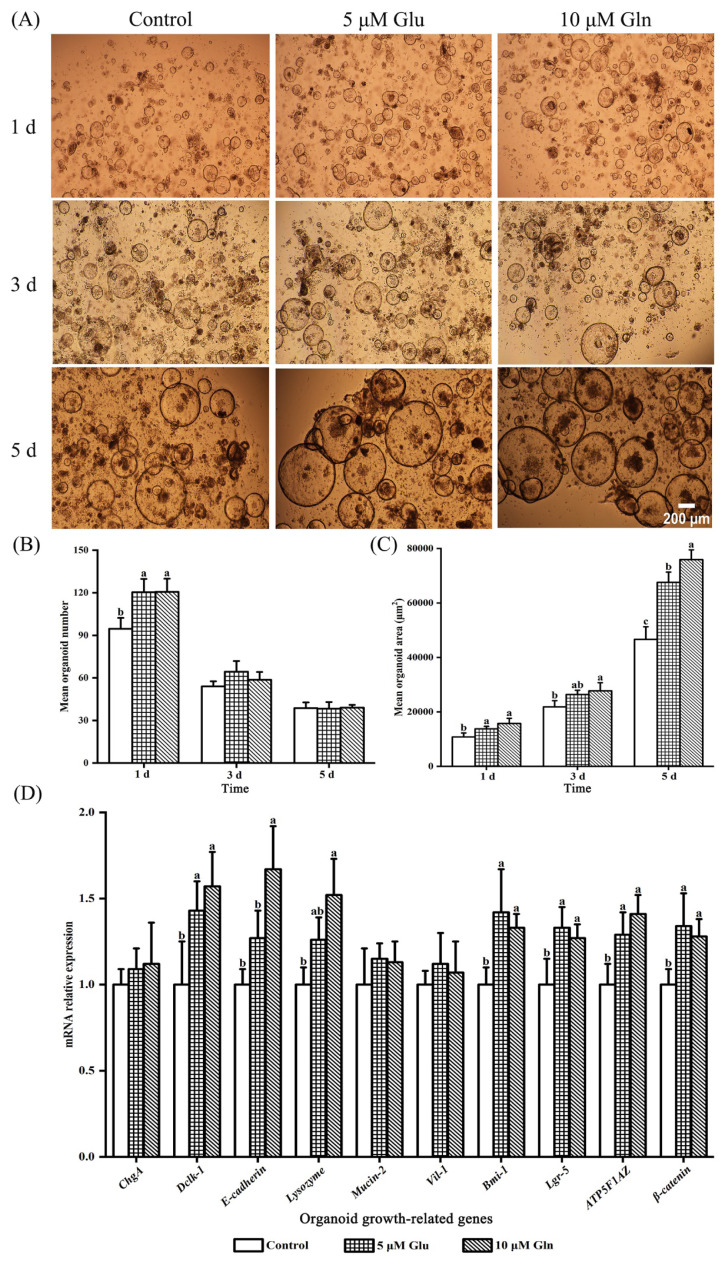
Effects of appropriate Glu and Gln dosages on the development and gene abundances of intestinal organoids. The mean of 3 replicates was used as the data. ^a–c^ Without any common superscripts, values amongst groups on the same day of age differ significantly (p<0.05), and the standard deviation (SD) is shown by the error bars. Glu, glutamic acid; Gln, glutamine.

The elevated values of the mean organoid number (1 d) and the mean organoid area (1 and 5 d) were observed in the 5 μM Glu and 10 μM Gln groups compared with the control group, as well as the mean organoid area in the 10 μM Gln group at 3 d (p<0.05; Figures 6A–6C). Additionally, the mean organoid area of the 10 μM Gln group was higher than that of the 5 μM Glu group at 5 d (p<0.05; Figure 6C). As shown in Figure 6D, no substantial changes were noted in the mRNA relative expressions of *ChgA*, *Mucin-2*, and *Vil-1* among all groups. Compared to the control group, the mRNA relative expressions of *Dclk-1*, *ATP5F1AZ*, *β-catenin*, *Bmi-1*, and *Lgr-5* increased in the 5 μM Glu and 10 μM Gln groups (p<0.05). Meanwhile, compared to the control group, higher values of the gene mRNA relative expressions of *E-cadherin* and *Lysozyme* were observed in the 10 μM Gln group (p<0.05), but there were no substantial changes in the 5 μM Glu group. Furthermore, the mRNA relative expression of *E-cadherin* enhanced in the 10 μM Gln treatment compared with the 5 μM Glu treatment (p<0.05). Additionally, as shown in Table 5, higher values of ATP content and Na^+^-K^+^-ATPase activity were observed in the 5 μM Glu and 10 μM Gln groups compared with the control group, and a higher value of Ca^2+^-Mg^2+^-ATPase activity was observed in the 10 μM Gln group (p<0.05). Furthermore, there were no differences in Na^+^-K^+^-ATPase activity and Ca^2+^-Mg^2+^-ATPase activity between the Glu and Gln groups, and the ATP content of intestinal organoids increased in the Gln group compared with the Glu group (p<0.05).

## DISCUSSION

Glu and Gln have been demonstrated to positively impact intestinal development and health status in various species, including pigs and chickens [23,24]. Nonetheless, several findings still suggest that higher dosages of Glu and Gln do not yield noticeable benefits in terms of the animal intestine [10,25]. Therefore, it is necessary to determine the optimal supplementation dosages during the chick stage of laying chickens. Based on our unpublished data (Supplements 1–4), dietary supplementation with 0.05% Glu and 0.20% Gln resulted in better growth performance and intestinal development than those of the control group. Thus, these 2 dosages for Glu and Gln were selected for this work. Besides, it is noteworthy that the lower dosage of Glu compared with Gln could be attributed to its nature as an acidic amino acid, where excessive amounts may negatively impact osmotic pressure and homeostasis [12].

Early intestinal development in chicks is susceptible to various factors (such as inadequate energy supply and diseases), which can lead to intestinal dysplasia and cause irreversible impacts on the subsequent growth and productivity of laying hens. Therefore, this study further investigated the effects of supplementing with appropriate dosages of Glu and Gln on growth performance and intestinal injury repair in layer chicks. An effective model of intestinal damage was established in this work by injecting LPS intraperitoneally, as evidenced by the fact that BW, ADG, and ADFI significantly decreased after LPS administration. This was in line with the previous study showing that intestinal damage models could be established through LPS injection in broilers [26]. Furthermore, the results of growth performance indicated that intraperitoneal injection of LPS in layer chicks caused a decrease in growth performance, which could be effectively mitigated by supplementing the diet with 0.05% Glu and 0.20% Gln. This was consistent with previous reports that supplementation with Glu and Gln could effectively improve growth performance [17,27]. Based on these findings, dietary supplementation with 0.05% Glu and 0.20% Gln effectively restored the reduction in growth performance of layer chicks caused by LPS. Additionally, at the end of the trial, the ultimate BW and ADG in the Gln treatment were significantly higher than those in the Glu treatment, which may be attributed to better intestinal development.

Intestinal development and growth performance are closely associated. Dietary supplementation with 0.05% Glu and 0.20% Gln improved LPS-impaired growth performance of the chicks, which may be attributed to their beneficial effects on small intestinal morphology and development [28]. Therefore, further research was conducted to investigate the impact of Glu and Gln on intestinal development and damage repair. The significant reductions in the weight and length of 3 intestinal segments in layer chicks demonstrated that LPS injection impeded the development of the small intestine. In this work, compared to the LPS treatment, dietary supplementation with Glu and Gln significantly improved the length, weight, and index of 3 intestinal segments in chicks that received LPS injection and offset the adverse impact of LPS administration on intestinal development. Additionally, the ileum parameters in the Gln group were higher compared with those in the Glu group after receiving LPS injection. Consistent with other reports, these findings suggested that Glu and Gln positively impacted intestine development, which may be attributed to improved intestinal morphology [9]. Based on the above analyses, the intestinal development of layer chicks was improved by dietary supplementation with 0.05% Glu and 0.20% Gln, and supplementation with 0.20% Gln had greater positive effects on ileum development than that of 0.05% Glu supplementation.

This study further investigated intestinal morphology, as healthy intestinal development is typically accompanied by favorable intestinal morphology. Notably, the injection of LPS impaired the morphology of the small intestine, as evidenced by the significant reduction in VH and VCR in this study. This finding was consistent with a previous report that LPS injection caused intestinal morphology damage in broilers [29]. Dietary supplementation with 0.05% Glu and 0.20% Gln significantly improved intestinal VH and VCR in 3 segments and ameliorated LPS-induced morphological damage. These results were in line with previous research showing that intestinal morphology was significantly enhanced by Glu and Gln [7,17,23]. Moreover, compared with 0.05% Glu addition, the values of all parameters numerically increased by 0.20% Gln addition, which may be attributed to the enhanced gene abundances of intestinal epithelial functional cells and stem cell marker genes [30]. All of these results indicated that early intestinal development and morphology in layer chicks could be improved by dietary supplementation with 0.05% Glu and 0.20% Gln, which could help alleviate the intestinal damage caused by LPS.

The maintenance of the intestinal mucosal structure depends on the growth and replacement of the intestinal epithelium [31], which are prerequisites and guarantees for forming a favorable intestinal morphology. The constant renewal and proliferation of IESCs are necessary for maintaining the homeostasis of the intestinal epithelium, among which there are two types of IESCs: fast-cycling IESCs and quiescent IESCs marked by Lgr-5 [32] and Bmi-1 [33], respectively. Lgr-5-marked fast-cycling IESCs could generate all epithelial cell types of mature intestinal epithelium, such as tuft cells, goblet cells, enteroendocrine cells, absorptive enterocytes, and Paneth cells [34,35]. Furthermore, Bmi-1-marked quiescent IESCs can generate fast-cycling IESCs marked by Lgr-5 under stressful circumstances [36]. Therefore, in this research, the mRNA relative expressions of *E-cadherin*, *Vil-1*, *ChgA*, *Dclk-1*, *Lysozyme*, *Mucin-2*, *Lgr-5*, and *Bmi-1* genes were measured, which were expected to act as the markers for the epithelium, absorptive enterocytes, enteroendocrine cells, tuft cells, Paneth cells, goblet cells, fast-cycling IESCs, and quiescent IESCs [19,37,38]. The results of gene abundance indicated that dietary supplementation with 0.05% Glu and 0.20% Gln could enhance the abundance of these genes, mitigating the reductions induced by LPS injection. This aligns with a previous study showing that dietary melatonin supplementation upregulated *Mucin-2* gene expression and counteracted the adverse effects of LPS administration [39]. Moreover, the benefits of 0.20% Gln addition on the gene abundances in the ileum were notably greater than those of 0.05% Glu supplementation treatment, which may be attributed to the superior energy metabolism [5]. According to these findings, the quantity of intestinal epithelial functional cells and stem cells could be enhanced by 0.05% Glu and 0.20% Gln addition treatments, and 0.20% Gln addition had better effects in the ileum than 0.05% Glu addition treatment.

Essential for maintaining epithelial integrity is the E-cadherin/*β*-catenin complex, and ATP synthase is responsible for the synthesis of ATP required by cells [40,41]. Furthermore, *β*-catenin is proposed to be necessary for the differentiation, proliferation, and renewal of stem cells [42], and energy metabolism is crucial for the maintenance and differentiation of stem cells [43]. Thus, the gene abundances (*ATP5F1AZ* and *β-catenin*) and energy metabolism levels were evaluated in this research. In this work, the gene abundances (*ATP5F1AZ* and *β-catenin*) and energy metabolism levels were negatively impacted by the administration of LPS. The notably higher gene abundances of intestinal epithelial functional cell and stem cell marker genes were previously observed in Glu and Gln addition treatments compared with the LPS group, which may be attributed to the significantly higher gene abundances (*ATP5F1AZ* and *β-catenin*) and energy metabolism levels than those of LPS treatment. These findings were similar to previous findings that intestinal epithelial functional cell and stem cell marker gene expressions increased in tandem with the gene abundance of *β-catenin* [44]. Additionally, compared to the Glu treatment, significantly higher values of ATP content, Na^+^-K^+^-ATPase activity, Ca^2+^-Mg^2+^-ATPase activity, and gene abundances (*ATP5F1AZ* and *β-catenin*) in the ileum were observed in the Gln supplemental treatment, which might confirm that Gln had better effects on the number of intestinal epithelial functional cells and stem cells in the ileum than those of the Glu supplemental treatment. Based on the above analysis, supplementation with 0.05% Glu and 0.20% Gln could promote the proliferation and differentiation of intestinal stem cells by improving energy metabolism. However, 0.20% Gln supplementation produced superior effects compared to 0.05% Glu.

To further verify the differences in the effects of Glu and Gln on the intestinal development of layer chicks, the intestinal organoid model was adopted in this work. Notably, no prior research has directly compared the effects of Glu and Gln supplementation on intestinal organoids. However, the application of intestinal organoids has promoted the research on disease pathogenesis, functional nutrients, and drug screening, for instance, in the pig species [30,45]. Therefore, in this study, intestinal organoids were cultured in the OGM supplemented with a range of Glu and Gln concentrations. Subsequently, 5 μM Glu and 10 μM Gln were selected to further investigate and compare their effects on intestinal organoid development at these appropriate concentrations. The results revealed that supplementation with 5 μM Glu and 10 μM Gln significantly improved the development of intestinal organoids compared to the control group. Furthermore, the final mean organoid area with 10 μM Gln supplementation was significantly greater than that observed with 5 μM Glu, which may be attributed to the higher number of functional cells and superior energy metabolism [44]. Thus, the gene abundances of growth-related genes and energy metabolism levels were measured. Compared with the control group, the gene abundances (*Dclk-1*, *ATP5F1AZ*, *β-catenin*, *Bmi-1*, and *Lgr-5*) and energy metabolism levels significantly increased in the Glu and Gln addition groups. These results indicated that 5 μM Glu and 10 μM Gln supplementation in the OGM might boost the development of intestinal organoids by stimulating the proliferation and differentiation of intestinal epithelial stem cells driven by energy supply [30]. Moreover, the gene abundances and energy metabolism levels of intestinal organoids were increased by OGM supplementation with Glu and Gln *in vitro*, which was consistent with the upregulation observed with dietary supplementation with Glu and Gln *in vivo*. Additionally, a significantly higher gene abundance of the epithelium marker gene *E-cadherin* was observed in the Gln treatment compared with the Glu treatment, which may be due to the significant rise in ATP content. Based on these findings, the mean area, gene abundance, and energy metabolism levels of intestinal organoids could be improved by 5 μM Glu and 10 μM Gln, among which 10 μM Gln had a greater effect.

## CONCLUSION

Dietary supplementation with 0.05% Glu and 0.20% Gln could alleviate the intestinal damage caused by LPS injection, as evidenced by improved intestinal development, intestinal morphology, and growth performance in layer chicks. Moreover, intestinal organoids derived from layer chicks and cultured in the OGM supplemented with 5 μM Glu and 10 μM Gln exhibited a significant increase in mean area and gene abundances compared with the control group. These findings could be attributed to the epithelial proliferation and differentiation, driven by improved energy metabolism caused by Glu and Gln supplementation. Furthermore, compared with 0.05% Glu supplementation, 0.20% Gln supplementation had a higher beneficial effect on growth performance, intestinal development, and intestinal injury repair in layer chicks, which might be attributed to higher energy metabolism levels.

## Figures and Tables

**Table 1:** Basic diet formula and nutrient level of layer chicks (air-dry basis)

Items	Contents (%)
Ingredient	
Corn	40.55
Soybean meal	26.90
Wheat bran	28.90
Calcium phosphate	1.00
Limestone	1.35
Soybean oil	1.00
DL-methionine	0.10
Salt	0.10
Premix^1)^	0.10
Nutrient level^2)^	
AME (MJ/kg)	11.71
Crude protein	20.14 (20.17)
Calcium	0.91 (0.9^2)^
Total phosphorus	0.77 (0.70)
Available phosphorus	0.40
Lysine	1.04
Methionine+cystine	0.75
Glutamic acid	(1.9^1)^
Glutamine	(1.44)

1) The following premix is provided per kg of diet: vitamin D3 4,125 IU, biotin 2 mg, Cu 11 mg, riboflavin 8.5 mg, Mn 51 mg, vitamin K3 2 mg, thiamine 1 mg, vitamin E 15 IU, I 0.51 mg, folic acid 5 mg, vitamin B12 5 mg, Fe 60 mg, Se 0.16 mg, Zn 65 mg, vitamin A 12,500 IU, pyridoxine 8 mg, niacin 32.5 mg, and Ca-pantothenate 50 mg.

2) The other nutrient levels are calculated values, and the levels in parentheses are analyzed values.

**Table 2:** Effects of dietary supplementation with Glu and Gln on the growth performance of layer chicks

Items^1)^	Control	LPS	LPS		SEM	p-value

			Glu	Gln		
BW (g)						
0 d	41.5	41.4	41.8	41.7	0.066	0.306
7 d	80.8^b^	80.6^b^	82.4^a^	82.8^a^	0.285	0.006
14 d	137^b^	126^c^	143^a^	145^a^	1.674	<0.001
21 d	210^c^	190^d^	219^b^	225^a^	2.865	<0.001
0 to 7 d						
ADG (g)	5.59^b^	5.60^b^	5.80^ab^	5.87^a^	0.041	0.016
ADFI (g)	11.8	11.6	11.8	11.8	0.065	0.679
FCR	2.11	2.08	2.04	2.00	0.016	0.089
7 to 14 d						
ADG (g)	8.02^b^	6.44^c^	8.66^a^	8.87^a^	0.222	<0.001
ADFI (g)	21.7^a^	19.3^b^	22.7^a^	22.3^a^	0.371	0.001
FCR	2.71^b^	3.01^a^	2.63^b^	2.52^b^	0.059	0.014
14 to 21 d						
ADG (g)	10.5^b^	9.19^c^	10.9^ab^	11.4^a^	0.215	<0.001
ADFI (g)	28.4	27.4	27.7	28.7	0.398	0.673
FCR	2.72^ab^	2.99^a^	2.57^b^	2.52^b^	0.067	0.043
0 to 21 d						
ADG (g)	8.03^c^	7.08^d^	8.44^b^	8.72^a^	0.135	<0.001
ADFI (g)	20.7^a^	19.4^b^	20.7^a^	20.9^a^	0.216	0.049
FCR	2.57^b^	2.75^a^	2.46^b^	2.40^b^	0.038	0.002

The mean of 6 replicates, each with 10 birds, is used as the data.

1) Control, fed the basal diet; LPS, fed the basal diet and received LPS administration; Glu, fed the basal diet supplemented with 0.05% Glu and received LPS administration; Gln, fed the basal diet supplemented with 0.20% Gln and received LPS administration.

a–d Significant differences exist between means inside a row without a common superscript (p<0.05).

LPS, lipopolysaccharide; Glu, glutamic acid; Gln, glutamine; SEM, standard error of the mean; BW, body weight; ADG, average daily gain; ADFI, average daily feed intake; FCR, feed conversion ratio (feed:gain, g:g).

**Table 3:** Effects of dietary supplementation with Glu and Gln on the small intestine injury repair of layer chicks

Items^1)^	Control	LPS	LPS		SEM	p-value

			Glu	Gln		
7 d of age						
Duodenum						
Weight (g)	1.51	1.46	1.60	1.62	0.028	0.143
Index (%)	1.87	1.81	1.96	1.96	0.027	0.131
Length (cm)	11.65^b^	11.56^b^	12.15^ab^	12.38^a^	0.122	0.034
Jejunum						
Weight (g)	2.17^b^	2.21^b^	2.58^a^	2.67^a^	0.059	<0.001
Index (%)	2.68^b^	2.74^b^	3.14^a^	3.24^a^	0.060	<0.001
Length (cm)	22.63^bc^	22.14^c^	24.16^ab^	24.33^a^	0.314	0.016
Ileum						
Weight (g)	1.63^b^	1.53^b^	1.85^a^	1.96^a^	0.048	0.001
Index (%)	2.02^b^	1.90^b^	2.26^a^	2.38^a^	0.051	<0.001
Length (cm)	22.46^b^	22.24^b^	23.58^a^	24.06^a^	0.233	0.006
Total						
Weight (g)	5.31^b^	5.20^b^	6.04^a^	6.26^a^	0.124	<0.001
Index (%)	6.55^b^	6.45^b^	7.36^a^	7.58^a^	0.122	<0.001
Length (cm)	56.73^b^	55.94^b^	59.88^ab^	60.77^a^	0.634	0.007
14 d of age						
Duodenum						
Weight (g)	2.29^a^	2.00^b^	2.35^a^	2.42^a^	0.044	<0.001
Index (%)	1.67	1.59	1.64	1.67	0.016	0.265
Length (cm)	13.65^ab^	13.05^b^	13.84^a^	14.17^a^	0.133	0.014
Jejunum						
Weight (g)	3.21^b^	2.72^c^	3.46^a^	3.41^ab^	0.070	<0.001
Index (%)	2.35^a^	2.16^b^	2.42^a^	2.35^a^	0.029	0.006
Length (cm)	28.56^a^	26.01^b^	30.34^a^	29.87^a^	0.495	0.003
Ileum						
Weight (g)	2.26^b^	1.94^c^	2.39^b^	2.60^a^	0.058	<0.001
Index (%)	1.65^b^	1.54^c^	1.67^b^	1.79^a^	0.024	<0.001
Length (cm)	24.88^ab^	23.12^b^	25.54^a^	26.50^a^	0.401	0.013
Total						
Weight (g)	7.75^a^	6.66^b^	8.20^a^	8.43^a^	0.164	<0.001
Index (%)	5.67^a^	5.29^b^	5.73^a^	5.81^a^	0.057	0.001
Length (cm)	67.09^ab^	62.18^b^	69.72^a^	70.53^a^	0.936	0.002
21 d of age						
Duodenum						
Weight (g)	3.16^b^	2.74^c^	3.28^b^	3.52^a^	0.070	<0.001
Index (%)	1.50^ab^	1.45^b^	1.49^b^	1.57^a^	0.014	0.011
Length (cm)	15.26^ab^	14.52^b^	15.76^a^	16.10^a^	0.196	0.015
Jejunum						
Weight (g)	4.30^b^	3.56^c^	4.39^b^	4.79^a^	0.104	<0.001
Index (%)	2.05^ab^	1.88^c^	2.00^b^	2.13^a^	0.027	0.002
Length (cm)	29.59^b^	27.49^c^	32.04^a^	30.83^ab^	0.480	0.001
Ileum						
Weight (g)	2.63^b^	2.24^c^	2.74^b^	3.08^a^	0.077	<0.001
Index (%)	1.25^b^	1.18^b^	1.25^b^	1.37^a^	0.022	0.012
Length (cm)	26.28^b^	23.94^c^	27.19^b^	29.42^a^	0.530	<0.001
Total						
Weight (g)	10.09^b^	8.53^c^	10.41^b^	11.39^a^	0.240	<0.001
Index (%)	4.80^b^	4.50^c^	4.73^bc^	5.06^a^	0.051	<0.001
Length (cm)	71.13^ab^	65.95^b^	74.99^a^	76.35^a^	1.121	<0.001

The mean of 6 replicates, each value averaged from 2 birds, is used as the data.

1) Control, fed the basal diet; LPS, fed the basal diet and received LPS administration; Glu, fed the basal diet supplemented with 0.05% Glu and received LPS administration; Gln, fed the basal diet supplemented with 0.20% Gln and received LPS administration.

a–c Significant differences exist between means inside a row without a common superscript (p<0.05).

LPS, lipopolysaccharide; Glu, glutamic acid; Gln, glutamine; SEM, standard error of the mean.

**Table 4:** Effects of dietary supplementation with Glu and Gln on the energy metabolism in small intestinal mucosa of layer chicks (14 d of age)

Items^1)^	Control	LPS	LPS		SEM	p-value

			Glu	Gln		
ATP content (μmol/g protein)						
Duodenum	17.29	15.81	17.01	17.59	0.323	0.232
Jejunum	26.62^ab^	24.77^b^	28.30^a^	27.57^a^	0.480	0.042
Ileum	22.56^b^	18.98^c^	22.98^b^	26.13^a^	0.664	<0.001
Na^+^-K^+^-ATPase (U/mg protein)						
Duodenum	5.06	4.95	5.21	5.07	0.086	0.804
Jejunum	4.07^b^	3.39^c^	4.61^a^	4.25^ab^	0.120	<0.001
Ileum	3.99^b^	3.44^c^	4.19^b^	4.89^a^	0.137	<0.001
Ca2^+^-Mg2^+^-ATPase (U/mg protein)						
Duodenum	4.95	4.67	5.18	5.11	0.087	0.159
Jejunum	4.06^b^	3.30^c^	4.74^a^	4.38^ab^	0.134	<0.001
Ileum	4.01^bc^	3.67^c^	4.11^b^	4.65^a^	0.098	<0.001

The mean of 6 replicates is used as the data.

1) Control, fed the basal diet; LPS, fed the basal diet and received LPS administration; Glu, fed the basal diet supplemented with 0.05% Glu and received LPS administration; Gln, fed the basal diet supplemented with 0.20% Gln and received LPS administration.

a–c Significant differences exist between means inside a row without a common superscript (p<0.05).

LPS, lipopolysaccharide; Glu, glutamic acid; Gln, glutamine; SEM, standard error of the mean.

**Table 5:** Effects of Glu and Gln supplementation in the OGM on the energy metabolism of intestinal organoids

Items^1)^	Control	Glu	Gln	SEM	p-value
ATP content (μmol/g protein)	2.39^c^	3.70^b^	4.83^a^	0.373	0.001
Na^+^-K^+^-ATPase (U/mg protein)	3.64^b^	4.88^a^	5.47^a^	0.304	0.011
Ca^2+^-Mg^2+^-ATPase (U/mg protein)	3.42^b^	4.01^ab^	4.70^a^	0.226	0.035

The mean of 3 replicates is used as the data.

1) Control, OGM was used for culture; Glu, OGM supplemented with 5 μM glutamic acid was used for culture; Gln, OGM supplemented with 10 μM glutamine was used for culture.

a–c Significant differences exist between means inside a row without a common superscript (p<0.05).

Glu, glutamic acid; Gln, glutamine; OGM, organoid growth medium; SEM, standard error of the mean.

## SUPPLEMENTARY MATERIAL

**Supplement 1**. Effects of Glu supplementation on the growth performance of layer chicks injected with LPS.

**Supplement 2**. Effects of Gln supplementation on the growth performence of layer chicks injected with LPS.

**Supplement 3**. Effects of Glu supplementation on the intestinal development of layer chicks injected with LPS.

**Supplement 4**. Effects of Gln supplementation on the intestinal development of layer chicks injected with LPS.

**Supplement 5**. Primers of intended and reference genes for layer chicks.

**Supplement 6**. Formulation and preparation of an organoid growth medium.

**Supplement 7**. The methods of immunofluorescence and 5-ethynyl-2′-deoxyuridine (EdU) labeling.
