# Supplementary material for: Comparative study on the effects of glutamic acid and glutamine in promoting intestinal development in chicks through energy metabolism
Source: Anim Biosci. 2025 Sep 30;39(2):250445. doi: 10.5713/ab.25.0445 (PMC12877385; doi:10.5713/ab.25.0445)
Supplement: Supplementary file 1 [file ab-25-0445-Supplementary-1.pdf]

Supplement 1. Effects of Glu supplementation on the growth performance of layer chicks injected with LPS

Items[1)]	Control	Glu dosages				SEM	p-value
		0.05%	0.10%	0.20%	0.40%		
BW, g							
0 d	41.5	41.8	41.8	41.6	41.7	0.047	0.429
7 d	80.8^{ab}	82.4^{a}	81.3^{ab}	80.5^{b}	80.3^{b}	0.224	0.019
14 d	137^{bc}	143^{a}	140^{ab}	138^{abc}	134^{c}	0.750	<0.001
21 d	210^{bc}	219^{a}	215^{ab}	205^{cd}	200^{d}	1.439	<0.001
0 to 7 d							
ADG, g	5.59	5.80	5.62	5.54	5.56	0.031	0.051
ADFI, g	11.8	11.8	11.8	11.8	11.9	0.063	0.953
FCR	2.11^{ab}	2.04^{b}	2.10^{ab}	2.13^{ab}	2.18^{a}	0.015	0.031
7 to 14 d							
ADG, g	8.02^{ab}	8.66^{a}	8.44^{a}	8.23^{ab}	7.71^{b}	0.091	0.004
ADFI, g	21.7^{b}	22.7^{ab}	22.6^{ab}	23.6^{a}	23.7^{a}	0.227	0.023
FCR	2.71^{b}	2.63^{b}	2.68^{b}	2.87^{ab}	3.09^{a}	0.041	<0.001
14 to 21 d							
ADG, g	10.5^{ab}	10.9^{a}	10.6^{a}	9.5^{bc}	9.3^{c}	0.158	<0.001
ADFI, g	28.4	27.7	29.1	29.6	28.9	0.408	0.678
FCR	2.72^{ab}	2.57^{b}	2.73^{ab}	3.12^{a}	3.12^{a}	0.068	0.015
0 to 21 d							
ADG, g	8.03^{bc}	8.44^{a}	8.24^{ab}	7.77^{cd}	7.52^{d}	0.068	<0.001
ADFI, g	20.7	20.7	21.2	21.7	21.5	0.188	0.347
FCR	2.57^{bc}	2.46^{c}	2.57^{c}	2.79^{ab}	2.86^{a}	0.036	<0.001

The mean of 6 replicates, each with 10 birds, is used as the data.

[1)] Control = fed the basal diet; Glu dosages = fed the basal diet supplemented with Glu (0.05%, 0.10%, 0.20% and 0.40%, respectively) and received LPS administration.

$^{a\text{-}d}$ Significant differences exist between means inside a row without a common superscript ($p<0.05$).

Glu, glutamic acid; LPS, lipopolysaccharide; BW, body weight; ADG, average daily gain; ADFI, average daily feed intake; FCR, feed conversion ratio (feed:gain, g:g); SEM, standard error of the mean.
