# Supplementary material for: Comparative study on the effects of glutamic acid and glutamine in promoting intestinal development in chicks through energy metabolism
Source: Anim Biosci. 2025 Sep 30;39(2):250445. doi: 10.5713/ab.25.0445 (PMC12877385; doi:10.5713/ab.25.0445)
Supplement: Supplementary file 2 [file ab-25-0445-Supplementary-2.pdf]

Supplement 2. Effects of Gln supplementation on the growth performance of layer chicks injected with LPS

Items[1)]	Control	Gln dosages				SEM	p-value
		0.20%	0.40%	0.80%	1.60%		
BW, g							
0 d	41.5	41.7	41.6	41.5	41.6	0.046	0.809
7 d	80.8^{ab}	82.8^{a}	80.9^{ab}	80.9^{ab}	80.1^{b}	0.253	0.009
14 d	137^{b}	145^{a}	138^{b}	141^{ab}	139^{b}	0.748	0.001
21 d	210^{c}	225^{a}	215^{bc}	218^{ab}	207^{c}	1.377	<0.001
0 to 7 d							
ADG, g	5.59^{b}	5.87^{a}	5.60^{b}	5.62^{ab}	5.51^{b}	0.035	0.007
ADFI, g	11.8	11.8	11.8	11.8	11.8	0.048	0.985
FCR	2.11^{a}	2.00^{b}	2.10^{ab}	2.09^{ab}	2.15^{a}	0.013	0.005
7 to 14 d							
ADG, g	8.02^{b}	8.87^{a}	8.09^{ab}	8.63^{ab}	8.47^{ab}	0.099	0.019
ADFI, g	21.7	22.3	21.4	22.1	22.2	0.200	0.597
FCR	2.71	2.52	2.64	2.56	2.63	0.029	0.276
14 to 21 d							
ADG, g	10.5^{ab}	11.4^{a}	10.8^{ab}	11.0^{ab}	9.72^{b}	0.168	0.010
ADFI, g	28.4	28.7	28.8	29.1	28.7	0.295	0.975
FCR	2.72	2.52	2.61	2.66	2.96	0.053	0.078
0 to 21 d							
ADG, g	8.03^{c}	8.72^{a}	8.27^{bc}	8.43^{ab}	7.90^{c}	0.065	<0.001
ADFI, g	20.7	20.9	20.6	21.0	20.9	0.144	0.929
FCR	2.57^{ab}	2.40^{b}	2.50^{ab}	2.49^{ab}	2.65^{a}	0.025	0.013

The mean of 6 replicates, each with 10 birds, is used as the data.

[1)] Control = fed the basal diet; Gln dosages = fed the basal diet supplemented with Gln (0.20%, 0.40%, 0.80% and 1.60%, respectively) and received LPS administration.

$^{a-c}$ Significant differences exist between means inside a row without a common superscript ($p<0.05$).

Gln, glutamine; LPS, lipopolysaccharide; BW, body weight; ADG, average daily gain; ADFI, average daily feed intake; FCR, feed conversion ratio (feed:gain, g:g); SEM, standard error of the mean.
