# Supplementary material for: Comparative study on the effects of glutamic acid and glutamine in promoting intestinal development in chicks through energy metabolism
Source: Anim Biosci. 2025 Sep 30;39(2):250445. doi: 10.5713/ab.25.0445 (PMC12877385; doi:10.5713/ab.25.0445)
Supplement: Supplementary file 3 [file ab-25-0445-Supplementary-3.pdf]

Supplement 3. Effects of Glu supplementation on the intestinal development of layer chicks injected with LPS

Items[1)]	Control	Glu dosages				SEM	p-value
		0.05%	0.10%	0.20%	0.40%		
7 d of age							
Duodenum							
Weight, g	1.51	1.60	1.51	1.41	1.42	0.027	0.129
Index, %	1.87	1.96	1.86	1.75	1.77	0.029	0.147
Length, cm	11.65	12.15	12.28	11.70	11.51	0.104	0.068
Jejunum							
Weight, g	2.17^{c}	2.58^{a}	2.50^{ab}	2.43^{abc}	2.23^{bc}	0.043	0.003
Index, %	2.68^{c}	3.14^{a}	3.08^{ab}	2.99^{abc}	2.79^{bc}	0.045	<0.001
Length, cm	22.63^{b}	24.16^{a}	22.78^{b}	22.36^{b}	22.38^{b}	0.219	0.041
Ileum							
Weight, g	1.63	1.85	1.70	1.62	1.64	0.030	0.066
Index, %	2.02	2.26	2.10	2.00	2.06	0.032	0.074
Length, cm	22.46	23.58	23.03	22.49	22.61	0.154	0.083
Total							
Weight, g	5.31^{b}	6.04^{a}	5.72^{ab}	5.46^{ab}	5.29^{b}	0.087	0.018
Index, %	6.55^{b}	7.36^{a}	7.04^{ab}	6.74^{ab}	6.61^{b}	0.087	0.009
Length, cm	56.73^{ab}	59.88^{a}	58.08^{ab}	56.55^{b}	56.42^{b}	0.412	0.023
14 d of age							
Duodenum							
Weight, g	2.29^{a}	2.35^{a}	2.28^{a}	2.15^{ab}	1.91^{b}	0.040	<0.001
Index, %	1.67^{a}	1.64^{a}	1.65^{a}	1.55^{ab}	1.42^{b}	0.024	<0.001
Length, cm	13.65	13.83	13.52	13.83	13.44	0.097	0.637
Jejunum							
Weight, g	3.21^{ab}	3.46^{a}	3.09^{b}	2.94^{b}	2.88^{b}	0.052	<0.001
Index, %	2.35^{ab}	2.42^{a}	2.25^{ab}	2.12^{b}	2.14^{b}	0.033	0.006
Length, cm	28.56^{ab}	30.34^{a}	28.74^{ab}	26.39^{b}	27.11^{b}	0.410	0.013
Ileum							
Weight, g	2.26^{ab}	2.39^{a}	2.20^{ab}	2.02^{b}	1.99^{b}	0.039	0.002
Index, %	1.65^{ab}	1.67^{a}	1.59^{ab}	1.47^{b}	1.48^{b}	0.024	0.008
Length, cm	24.88	25.54	25.06	23.45	23.21	0.328	0.080
Total							
Weight, g	7.75^{ab}	8.20^{a}	7.56^{ab}	7.11^{bc}	6.78^{c}	0.113	<0.001
Index, %	5.67^{a}	5.73^{a}	5.49^{ab}	5.15^{bc}	5.03^{c}	0.065	<0.001
Length, cm	67.09^{ab}	69.72^{a}	67.32^{ab}	63.68^{b}	63.76^{b}	0.697	0.015
21 d of age							
Duodenum							
Weight, g	3.16^{ab}	3.28^{a}	3.11^{ab}	2.93^{b}	2.59^{c}	0.055	<0.001
Index, %	1.50^{a}	1.49^{a}	1.45^{a}	1.44^{a}	1.33^{b}	0.016	<0.001
Length, cm	15.26	15.76	15.31	15.42	14.50	0.146	0.078
Jejunum							
Weight, g	4.30^{a}	4.39^{a}	4.18^{a}	3.71^{b}	3.35^{b}	0.084	<0.001
Index, %	2.05^{a}	2.00^{ab}	1.95^{ab}	1.83^{bc}	1.72^{c}	0.029	<0.001
Length, cm	29.59^{ab}	32.04^{a}	30.18^{ab}	27.78^{b}	27.59^{b}	0.441	0.002
Ileum							
Weight, g	2.63^{ab}	2.74^{a}	2.62^{ab}	2.41^{bc}	2.16^{c}	0.050	<0.001
Index, %	1.25^{a}	1.25^{a}	1.22^{ab}	1.19^{ab}	1.11^{b}	0.016	0.018
Length, cm	26.28^{ab}	27.19^{a}	26.74^{a}	25.24^{ab}	23.61^{b}	0.370	0.008
Total							
Weight, g	10.09^{a}	10.41^{a}	9.91^{ab}	9.05^{b}	8.10^{c}	0.180	<0.001
Index, %	4.80^{a}	4.73^{ab}	4.62^{ab}	4.46^{bc}	4.16^{c}	0.053	<0.001
Length, cm	71.13^{abc}	74.99^{a}	72.23^{ab}	68.44^{bc}	65.70^{c}	0.854	0.002

The mean of 6 replicates, each value averaged from 2 birds, is used as the data.

[1)] Control = fed the basal diet; Glu dosages = fed the basal diet supplemented with Glu (0.05%, 0.10%, 0.20% and 0.40%, respectively) and received LPS administration.

[a-c] Significant differences exist between means inside a row without a common superscript ($p<0.05$).

Glu, glutamic acid; LPS, lipopolysaccharide; SEM, standard error of the mean.
