# Supplementary material for: Comparative study on the effects of glutamic acid and glutamine in promoting intestinal development in chicks through energy metabolism
Source: Anim Biosci. 2025 Sep 30;39(2):250445. doi: 10.5713/ab.25.0445 (PMC12877385; doi:10.5713/ab.25.0445)
Supplement: Supplementary file 4 [file ab-25-0445-Supplementary-4.pdf]

Supplement 4. Effects of Gln supplementation on the intestinal development of layer chicks injected with LPS

Items[1)]	Control	Gln dosages				SEM	p-value
		0.20%	0.40%	0.80%	1.60%		
7 d of age							
Duodenum							
Weight, g	1.51	1.62	1.48	1.58	1.42	0.028	0.157
Index, %	1.87^{ab}	1.96^{a}	1.82^{ab}	1.94^{ab}	1.76^{b}	0.024	0.037
Length, cm	11.65	12.38	11.68	11.82	11.41	0.121	0.110
Jejunum							
Weight, g	2.17^{b}	2.67^{a}	2.26^{b}	2.43^{ab}	2.36^{ab}	0.052	0.013
Index, %	2.68^{b}	3.24^{a}	2.80^{b}	2.98^{ab}	2.92^{ab}	0.055	0.007
Length, cm	22.63	24.33	22.73	23.29	22.76	0.226	0.085
Ileum							
Weight, g	1.63^{b}	1.96^{a}	1.67^{ab}	1.78^{ab}	1.59^{b}	0.039	0.008
Index, %	2.02^{b}	2.38^{a}	2.07^{ab}	2.19^{ab}	1.97^{b}	0.044	0.015
Length, cm	22.46	24.06	22.76	23.19	23.03	0.190	0.076
Total							
Weight, g	5.31^{b}	6.26^{a}	5.40^{b}	5.78^{ab}	5.36^{b}	0.102	0.006
Index, %	6.55^{b}	7.58^{a}	6.69^{b}	7.12^{ab}	6.65^{b}	0.099	<0.001
Length, cm	56.73^{b}	60.77^{a}	57.16^{ab}	58.30^{ab}	57.19^{ab}	0.479	0.039
14 d of age							
Duodenum							
Weight, g	2.29	2.42	2.32	2.35	2.26	0.025	0.356
Index, %	1.67	1.67	1.70	1.66	1.62	0.015	0.643
Length, cm	13.65	14.17	13.89	13.75	13.84	0.089	0.453
Jejunum							
Weight, g	3.21	3.41	3.13	3.11	3.25	0.048	0.293
Index, %	2.35	2.35	2.29	2.19	2.33	0.025	0.224
Length, cm	28.56	29.87	28.88	28.44	28.93	0.303	0.631
Ileum							
Weight, g	2.26^{b}	2.60^{a}	2.35^{ab}	2.24^{b}	2.29^{b}	0.036	0.004
Index, %	1.65^{bc}	1.79^{a}	1.72^{ab}	1.58^{c}	1.64^{bc}	0.018	<0.001
Length, cm	24.88	26.50	25.47	25.23	25.13	0.265	0.358
Total							
Weight, g	7.75	8.43	7.81	7.71	7.81	0.096	0.086
Index, %	5.67^{ab}	5.81^{a}	5.70^{ab}	5.43^{b}	5.58^{ab}	0.042	0.047
Length, cm	67.09	70.53	68.24	67.42	67.89	0.582	0.377
21 d of age							
Duodenum							
Weight, g	3.16^{b}	3.52^{a}	3.25^{ab}	3.30^{ab}	3.20^{b}	0.037	0.012
Index, %	1.50	1.57	1.51	1.51	1.54	0.010	0.224
Length, cm	15.26	16.10	15.49	15.54	15.20	0.146	0.325
Jejunum							
Weight, g	4.30^{b}	4.79^{a}	4.51^{ab}	4.41^{b}	4.21^{b}	0.052	0.001
Index, %	2.05	2.13	2.09	2.02	2.03	0.015	0.084
Length, cm	29.59	30.83	29.89	29.60	29.39	0.262	0.455
Ileum							
Weight, g	2.63^{b}	3.08^{a}	2.90^{ab}	2.71^{ab}	2.73^{ab}	0.050	0.023
Index, %	1.25	1.37	1.35	1.24	1.31	0.019	0.079
Length, cm	26.28	29.42	27.48	27.19	26.95	0.389	0.108
Total							
Weight, g	10.09^{b}	11.39^{a}	10.66^{ab}	10.42^{b}	10.14^{b}	0.123	0.001
Index, %	4.80^{b}	5.06^{a}	4.95^{ab}	4.77^{b}	4.89^{ab}	0.031	0.009
Length, cm	71.13	76.35	72.86	72.33	71.54	0.715	0.145

The mean of 6 replicates, each value averaged from 2 birds, is used as the data.

[1)] Control = fed the basal diet; Gln dosages = fed the basal diet supplemented with Gln (0.20%, 0.40%, 0.80% and 1.60%, respectively) and received LPS administration.

[a-c] Significant differences exist between means inside a row without a common superscript ($p<0.05$).

Gln, glutamine; LPS, lipopolysaccharide; SEM, standard error of the mean.
