# Supplementary material for: Comparative study on the effects of glutamic acid and glutamine in promoting intestinal development in chicks through energy metabolism
Source: Anim Biosci. 2025 Sep 30;39(2):250445. doi: 10.5713/ab.25.0445 (PMC12877385; doi:10.5713/ab.25.0445)
Supplement: Supplementary file 5 [file ab-25-0445-Supplementary-5.pdf]

Supplement 5. Primers of intended and reference genes for layer chicks

Gene	Primer sequence (5’-3’)	GenBank number	Length (bp)
ChgA[1)]	F: TGAATAAAGGGGACACTAAGG<br>R: AGCTCAGCCAGGGATG		337
Dclk-1	F: AACTGCCACGTGAAAGTCCT<br>R: AGGGTTTGGTCCCATGAACA	XM_015277813.4	196
E-cadherin[1)]	F: ACTGGTGACATTATTACCGTAGCA<br>R: TAGCCACTATGACATCCACTCTGT	NM_001001615	226
Lysozyme[2)]	F: ATACAGCCTGGGAAACTGGGT<br>R: ATACAGCCTGGGAAACTGGGT	NM_205281.2	71
Mucin-2[2)]	F: TGTGGTCTGTGTGGCAACTT<br>R: GGCCTGAGCCTTGGTACATT	XM_046942297.1	357
Vil-1[2)]	F: CTACCTCTGCGGGGATGAGC<br>R: CTGTTGGCGTAGCTGGTCTT	NM_001396564.1	141
ATP5F1AZ	F: CCCTTGGTGCCGCATTTGTT<br>R: ACACACGGGCAATACCATCA	NM_204286.3	180
β-catenin	F: GTTCTTTCCACTCTGGCGGAT<br>R: ACTCCTCGACCAAAAAGGACC	NM_205081.3	374
Bmi-1[2)]	F: TTTCAAGATGGCCGCTTGGC<br>R: TGCACGTCTTGCAGAAGGAGT	NM_001007988.3	255
Lgr-5[2)]	F: CCTTTATCAGCCCAGAAGTGA<br>R: TGGAACAAATGCTACGGATG	XM_425441.4	338
β-actin[1)]	F: TGCTGTGTTCCCATCTATCG<br>R: TTGGTGACAATACCGTGTTCA	NM_205518.1	150

ChgA, chromogranin A; *Dclk-1*, doublecortin like kinase 1; *ATP5F1AZ*, ATP synthase F1 subunit alpha Z chromosome; *Bmi-1*, B-cell-specific Moloney murine leukemia virus insertion site 1; *Lgr-5*, leucine-rich-repeat-containing G-protein-coupled receptor 5; F, forward primer; R, reverse primer.

[1)] Sequences refer to Zhao et al. (2022).

[2)] Sequences refer to Cui et al. (2023).
