# Supplementary material for: Comparative study on the effects of glutamic acid and glutamine in promoting intestinal development in chicks through energy metabolism
Source: Anim Biosci. 2025 Sep 30;39(2):250445. doi: 10.5713/ab.25.0445 (PMC12877385; doi:10.5713/ab.25.0445)
Supplement: Supplementary file 6 [file ab-25-0445-Supplementary-6.pdf]

Supplement 6. Formulation and preparation of an organoid growth medium

The OGM contained DMEM/F-12 (Gibco), HEPES (10 mM, Solarbio), N-Acetyl-L-Cysteine (1 mM, Aladdin), EGF (50 ng/ml, Novoprotein), A8301 (500 nM, MCE), SB202190 (10 mM, MCE), Y-27632 (10 μM, MCE), PGE2 (0.02 μM, Adooq), GlutaMAX Supplement (1:100, Gibco), Penicillin-Streptomycin-Nystatin Solution (1:100, VivaCell), fetal bovine serum (10%, FBS, Zeta life), and 60% L-WRN conditioned medium [1]. According to the published protocol [2], L-WRN cells (RRID, CVCL_DA06) were cultured and the L-WRN conditioned medium was collected.

References

1. Zhao D, Farnell MB, Kogut MH, et al. From crypts to enteroids: establishment and characterization of avian intestinal organoids. Poult Sci 2022;101:101642. https://doi.org/10.1016/j.psj.2021.101642

2. Miyoshi H, Stappenbeck T S. *In vitro* expansion and genetic modification of gastrointestinal stem cells in spheroid culture. Nat Protoc 2013;8:2471-2482. https://doi.org/10.1038/nprot.2013.153
