# Supplementary material for: Comparative study on the effects of glutamic acid and glutamine in promoting intestinal development in chicks through energy metabolism
Source: Anim Biosci. 2025 Sep 30;39(2):250445. doi: 10.5713/ab.25.0445 (PMC12877385; doi:10.5713/ab.25.0445)
Supplement: Supplementary file 7 [file ab-25-0445-Supplementary-7.pdf]

Supplement 7. The methods of immunofluorescence and 5-ethynyl-2'-deoxyuridine (EdU) labeling

Immunofluorescence

The intestinal organoids were washed with TBSTw (Beyotime) and blocked with 10% goat serum (Bioss) at room temperature for 1 h, followed by overnight incubation at 4°C with E-Cadherin Mouse Monoclonal Antibody (1:200, Beyotime). After 3 washes with TBSTw, the secondary antibody Goat Anti-Mouse IgG/SAlexa Fluor 555 (1:1000, Solarbio) was added and incubated at room temperature for 1 h, then washed with TBSTw three times, and the cell nucleus was mounted with an Anti-fade mounting medium containing DAPI (Solarbio) to stain the nuclei. Organoid images were captured by an inverted fluorescence microscope (Discover Echo).

5-Ethynyl-2'-deoxyuridine (EdU) staining

After 5 d of culture, 50 μM EdU was added to the OGM and incubated for 2 h. The medium was discarded and the organoids were washed 3 times with PBS. Organoids were fixed at room temperature with 4% paraformaldehyde for 30 min, then organoids were treated with 2mg/mL glycine to neutralize the aldehyde groups for 5 min. The PBS was discarded after organoids were washed with PBS for 5 min. The organoids were incubated for 10 min with 0.5% Triton X-100 (Solarbio) in PBS, then washed with PBS for 5 min and discarded. The Cell-Light EdU Apollo 643 In Vivo Kit (Ribobio) was used to stain the organoids, and the cell nucleus was mounted with an Anti-fade mounting medium containing DAPI. Images were obtained by an inverted fluorescence microscope.
